# The Role of Cocaine- and Amphetamine-Regulated Transcript (CART) in Cancer: A Systematic Review

**DOI:** 10.3390/ijms24129986

**Published:** 2023-06-10

**Authors:** Maja Owe-Larsson, Jan Pawłasek, Tomasz Piecha, Alicja Sztokfisz-Ignasiak, Mikołaj Pater, Izabela R. Janiuk

**Affiliations:** 1 Department of Histology and Embryology, Center of Biostructure Research, Medical University of Warsaw, Chałubińskiego 5, 02-004 Warsaw, Poland; maja.owelarsson@onet.pl (M.O.-L.); janpawlasek1903@gmail.com (J.P.); alicja.sztokfisz-ignasiak@wum.edu.pl (A.S.-I.); mikolajpater@gmail.com (M.P.); 2 Department of General, Oncological and Functional Urology, Medical University of Warsaw, Lindleya 4, 02-005 Warsaw, Poland; tomasz.piecha@wum.edu.pl

**Keywords:** cocaine- and amphetamine-regulated transcript, tumor, marker, cell cycle, proliferation

## Abstract

The functions of cocaine- and amphetamine-regulated transcript (CART) neuropeptide encoded by the *CARTPT* gene vary from modifying behavior and pain sensitivity to being an antioxidant. Putative CART peptide receptor GPR160 was implicated recently in the pathogenesis of cancer. However, the exact role of CART protein in the development of neoplasms remains unclear. This systematic review includes articles retrieved from the Scopus, PubMed, Web of Science and Medline Complete databases. Nineteen publications that met the inclusion criteria and describe the association of CART and cancer were analyzed. CART is expressed in various types of cancer, e.g., in breast cancer and neuroendocrine tumors (NETs). The role of CART as a potential biomarker in breast cancer, stomach adenocarcinoma, glioma and some types of NETs was suggested. In various cancer cell lines, *CARTPT* acts an oncogene, enhancing cellular survival by the activation of the ERK pathway, the stimulation of other pro-survival molecules, the inhibition of apoptosis or the increase in cyclin D1 levels. In breast cancer, CART was reported to protect tumor cells from tamoxifen-mediated death. Taken together, these data support the role of CART activity in the pathogenesis of cancer, thus opening new diagnostic and therapeutic approaches in neoplastic disorders.

## 1. Introduction

Cocaine- and amphetamine-regulated transcript (CART) was discovered for the first time in 1995, when a previously uncharacterized mRNA was identified in the rat stratum [1]. Its relative expression levels were prominently increased by acute cocaine or amphetamine administration, hence the name CART [1,2]. Soon, it was clear that in different species including humans, CART peptide is widely distributed over multiple areas of the brain, including, e.g., the pituitary gland, hypothalamus, brain stem or dorsal horns of the spinal cord [3,4]. It is also present in neuroendocrine cells of the intestine [5], pancreas [6] and adrenal medulla [3], as well as in the gastrin-producing G-cells in the stomach [7].

**Figure 1:**
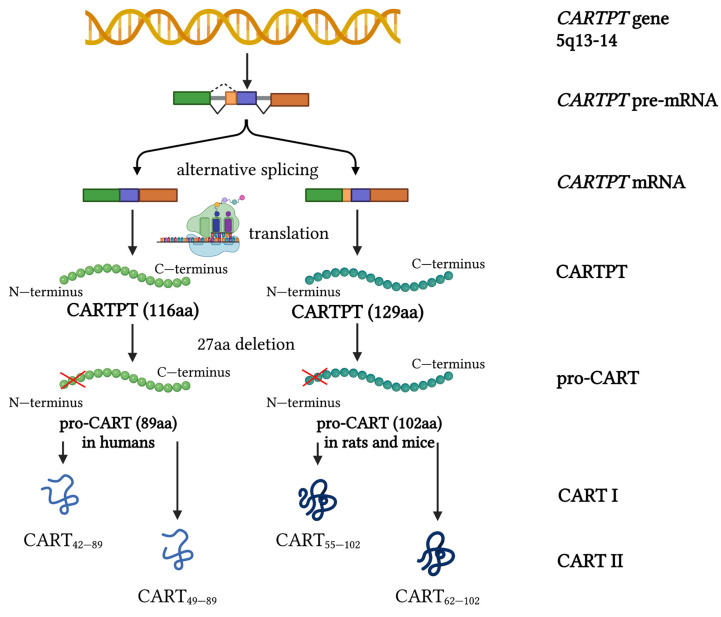
Schematic representation of the *CARTPT* transcripts and CART protein isoforms. aa—amino acids; CART—cocaine- and amphetamine-regulated transcript; *CARTPT*—cocaine- and amphetamine-regulated transcript prepropeptide. Red cross signifies deletion of 27 N—terminal amino acids. Created with BioRender.com, accessed on 9 June 2023.

CART peptide originates from CART prepropeptide (CARTPT) encoded by the *CARTPT* gene. It is a conserved gene with high interspecies homology [8]. There are three distinct exons and two introns of the *CARTPT* gene [8]. In humans, it is located on chromosome 5q13-14 [9]. The single primary *CARTPT* mRNA codes for two CARTPT molecules—129 amino acids (aa) and 116 aa, generated due to alternative splicing [1] (Figure 1). Their N-terminal hydrophobic signal sequence (27aa) is later deleted, leaving two pro-CART isoforms—long (102 aa) and short (89 aa). Only the short form is expressed in humans [10]. Pro-CART proteins are processed by prohormone convertases during their transport from the Golgi apparatus to mature secretory granules. Finally, the bioactive peptides CART I (CART_55–102_/CART_42–89_) and CART II (CART_62–102_/CART_49–89_) are produced. Both peptides can be generated from either the long or short pro-CART isoforms. CART_55–102_ and CART_62–102_ are present in rats and mice, while CART_42–89_ and CART_49–89_ are present in humans. Several other CART peptides also exist due to the presence of other cleavage sites in pro-CART proteins [11].

The most prominent effects of CART are related to modulation of behavior and brain function. CART peptide inhibits food intake, acts as a satiety signal and leads to weight loss in rats, but surprisingly, it can also be found in orexigenic neurons [12]. The anorexigenic activity of CART is modulated by leptin and neuropeptide Y [13]. CART also participates in stress modulation [14] and was implicated in the pathogenesis of psychiatric disorders such as depression or anxiety [15,16]. It may be also involved in the development of drug-dependency and addictions [17]. CART seems to be connected with hypersensitivity and neuropathic pain [18,19]. Furthermore, CART protein was reported to act as a strong antioxidant, neutralizing reactive oxygen species (ROS) [20]. ROS are able to damage cellular proteins, lipids and nucleic acids such as mitochondrial DNA (mtDNA). Thus, CART’s localization in mitochondria may have protective effects [20]. CART_55–102_ also increases the function of mitochondrial respiratory chain complex II and depletes the levels of ROS in neurons after oxygen–glucose deprivation [21].

Putative CART receptors were identified recently. CART was suggested to be a ligand of G-protein-coupled receptor 160 (GPR160), previously known as one of the orphan G-protein-coupled receptors [18]. Accumulating evidence suggests the association of GPR160 with cancer. In prostate cancer cells, the GPR160 knockdown increased expression of caspase 1 and interleukin 6, inhibited cell proliferation and evoked apoptosis [22]. Similarly, knockdown of GPR160 in glioma cells resulted in the promotion of apoptosis, decreased proliferation rate, reduced cell viability and diminished invasion ability [23]. In triple-negative breast cancer, GPR160 was found to be downregulated, and its higher level was correlated with better prognosis [24]. In contrast, amplification of GPR160 is connected with the development of nasopharyngeal carcinoma [25]. Additionally, in melanoma metastases, GPR160 expression was higher than in benign nevi [26]. Upregulated expression of GPR160 was also detected in CD4+CD56+ hematodermic neoplasm, termed blastic natural killer-cell lymphoma [27].

Signal transduction through GPR160 leads to the activation of extracellular signal-regulated kinase/cAMP response element-binding protein (ERK/CREB) pathway in rodents [18]. CART induces signal transduction through Gi/o-dependent protein kinase A (PKA)/ERK/CREB phosphorylation in rat nucleus accumbens and hypothalamic paraventricular nucleus [28], but it also stimulates the production of cyclic adenosine monophosphate (cAMP), which indicates the involvement of a Gsα-coupled receptor [29].

**Figure 2:**
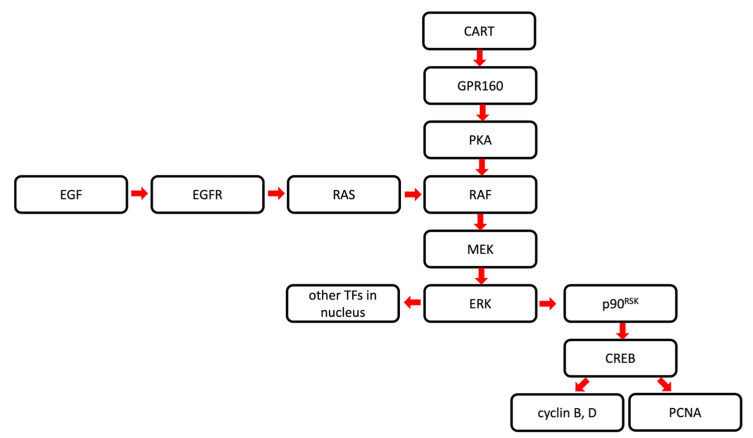
Schematic representation of the role of CART/GPR160 signaling in RAF/MEK/ERK pathway. CART—cocaine- and amphetamine-regulated transcript; CREB—cAMP response element-binding protein; EGF—epidermal growth factor; EGFR—epidermal growth factor receptor; ERK—extracellular signal-regulated kinase; GPR160—G-protein-coupled receptor 160; MEK—mitogen-activated protein kinase; p90RSK—90 kDa ribosomal S6 kinase; PCNA—proliferating cell nuclear antigen; PKA—protein kinase A; RAF—rapidly accelerated fibrosarcoma; RAS—rat sarcoma virus; TF—transcription factor.

ERK is a member of the mitogen-activated protein kinases (MAPK) family that plays a role in cell proliferation, differentiation, migration and apoptosis [30]. ERK1 and ERK2 take part in the activation of cells mostly via growth factors, such as epidermal growth factor (EGF). The binding of EGF to EGF receptors (EGFR) leads to the activation of rat sarcoma virus/rapidly accelerated fibrosarcoma/mitogen-activated protein kinase kinase/ERK (RAS/RAF/MEK/ERK) cascade. Phosphorylation of ERK1 and 2 results in their translocation to the nucleus and further activation of transcriptions factors, eventually leading to changes in specific gene expression. ERK was shown to activate via phosphorylation the 90 kDa ribosomal S6 kinase (p90^RSK^) [31], causing activation of CREB, another transcription factor, which participates in cell cycle regulation [32,33]. RAS and RAF mutations are commonly present in cancer cells and are important factors of tumorigenesis. MEK and ERK mutations are associated with worse prognosis in cancer patients; however, they are rather rare [30,34,35]. The putative roles of CART and GPR160 in the RAF/MEK/ERK pathway and regulation of cell growth and cancer progression are summarized in Figure 2.

The cell cycle is defined as the sequence of events occurring in a cell that enable its division and consists of four stages: G1, S, G2, and M. Its progression depends on at least 48 cell cycle proteins [36] and is regulated by cyclins, cyclin-dependent kinases (CDKs) and a number of kinases and transcription factors. Currently, about 30 different cyclins/cyclin-like proteins and around 20 different CDKs/CDK-like molecules have been identified [37]. Cell cycle checkpoints control the sequence, stability and accuracy of its key events, monitoring, among others, the DNA replication integrity, response to DNA damage, mitotic spindle attachment, chromosome segregation and cell size control [38]. Dysregulation of the cell cycle promotes carcinogenesis by facilitating continuous division [39]. CREB, a potential effector of CART, regulates cell proliferation by modulation of cyclin B, D and proliferating cell nuclear antigen (PCNA) expression in glioma cells [40].

In this qualitative systematic review, we provide a description of the current research focusing on the role of CART in oncologic diseases. We included reports published between 2000 and 2022. After excluding publications that did not meet the inclusion criteria, we analyzed the remaining ones. Then, the reports were categorized based on the type of analyzed material. The role of CART in the development of neoplasms and potential impact on tumor growth is discussed.

## 2. Methods

This systematic review of available literature concerning the associations of CART and cancer was performed in accordance with the preferred reporting items for systematic review and meta-analysis (PRISMA) 2020 statement guidelines [41]. The review was registered in OSF Registries with a number osf.io/6kq2g.

### 2.1. Literature Search

A comprehensive literature search was performed by three investigators (MOL, JP and IJ) in the following databases: PubMed, Medline Complete, Scopus and Web of Science, last accessed 8 August 2022. The search was limited to articles in English, published from the year 2000 up to the date of the search, without restrictions to publication type. Our search strategy included the keywords “Cocaine and amphetamine regulated transcript” OR “Cocaine- and amphetamine-regulated transcript” AND “neoplas*” OR “malignan*” OR “growth factor*” OR “cancer*” OR “GPR160”, in combinations with and without the abbreviation “CART” as a separate keyword. All results were compared between the investigators and were identical. In the results from databases PubMed, Medline Complete and Web of Science, the number of results did not differ depending on the presence or absence of dashes in the name “cocaine and amphetamine regulated transcript”, but in Scopus such a difference occurred, with a larger number of publications with dashes present. Adding the abbreviation “CART” limited the number of results in every database, and all publications found in the search with the abbreviation were also present in searches without it.

### 2.2. Inclusion and Exclusion Criteria

All relevant articles regarding the association of CART with cancer were screened by three reviewers (IJ, MOL and JP) after removing duplicates, based on the titles, abstracts, and full texts. Eligible studies met the following inclusion criteria: (1) the papers were original studies, (2) the used materials were either samples from human patients or cell lines (human or animal), (3) the publication was accessible, (4) it was published in English. The following types of publications were excluded: (1) reviews, case reports and meeting abstracts, (2) animal studies, (3) studies lacking results relevant to the subject of this paper, (4) publications with duplicated data.

A manual search was also performed in order to identify additional studies in the reference lists of the included publications. Results were described in a narrative manner, without meta-analysis of the data.

## 3. Results and Discussion

### 3.1. Results

**Figure 3:**
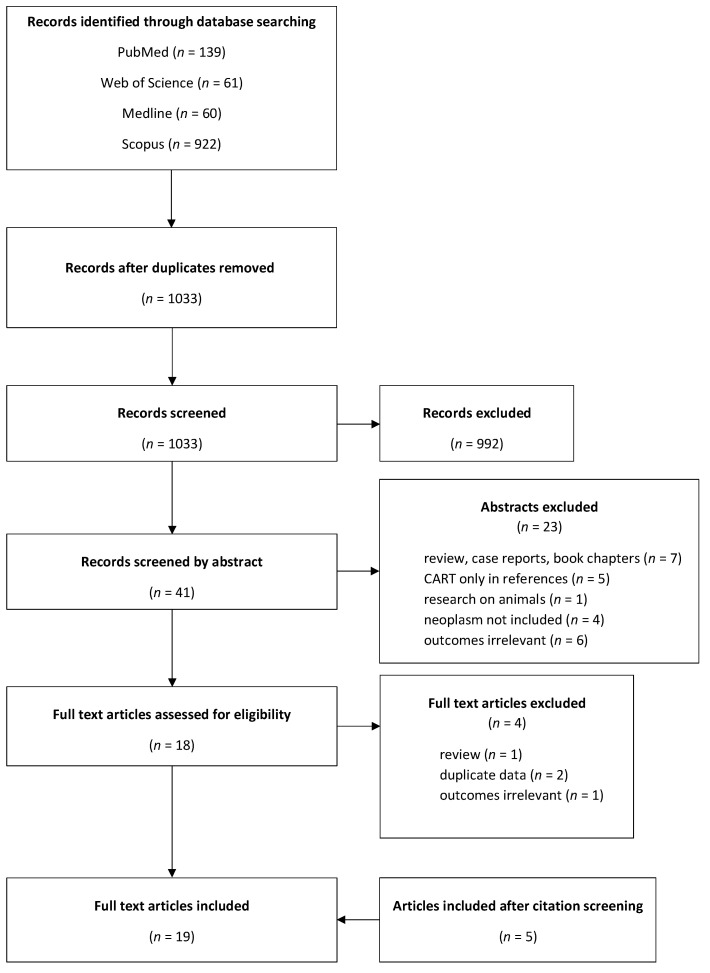
PRISMA flow diagram for identification of publications suitable for inclusion.

Overall, 1182 publications were retrieved (922 in Scopus, 139 in PubMed, 61 in Web of Science and 60 in Medline Complete). After removing duplicates, the number of publications equaled 1033, all of which were screened by title, leaving 41 studies. Nineteen reports were included after the reading of abstracts. Four full-text articles were excluded for the following reasons: the data were duplicated (two papers), the study was a review (one paper), and the paper concentrated only on identifying possible CART receptors (one paper). Five further studies were added after citation screening, leaving a total of nineteen full-text articles included in the review. A flow diagram is presented in Figure 3.

**Figure 4:**
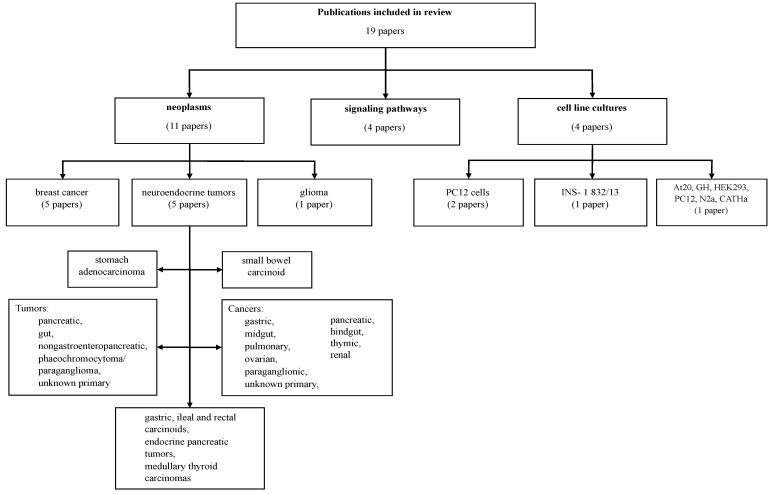
Cancer types and cell lines described in publications included in this review.

This review comprises 19 publications included after analyzing the full texts. The following cancer types were investigated: breast cancer (five papers), glioma (one paper), and various neuroendocrine tumors (five papers). Data on sample size, patient information and study design are provided in Table 1. Experiments were also performed on cell lines (four papers). Those 15 publications are described in Table 2. The other (four) papers that did not address the relationship between CART and neoplasms but described signaling pathways which were shown to be activated by CART are summarized in Table 3. Figure 4 provides detailed information concerning cancer types and cell lines as subject of the research in the included publications.

### 3.2. Discussion

#### 3.2.1. CART in Signaling Pathways

Uncontrolled cell proliferation resulting from cell cycle progression is one of the most important mechanisms in carcinogenesis and is promoted by certain signaling pathways, which induce RAF/MEK/ERK and phosphoinositide 3-kinase/3′-phosphoinositide-dependent kinase/protein kinase B (PI3K/PDK/PKB = Akt) pathways. These pathways promote G0 → G1 → S-phase cell cycle progression by induction of cyclin D1 and repression of cyclin-dependent kinase inhibitor 1B (p27^Kip1^) expression. Akt kinase also causes continuous activation of cyclin/CDK2 complexes and removal of RAF-induced cyclin-dependent kinase inhibitor 1 (p21^Cip1^) from complexes of cyclin E/CDK2 [42].

CART may influence these signaling pathways, since it activates Akt (demonstrated in INS-1(832/13) cells) [43], furthermore, CART_55–102_ enhances ERK phosphorylation (as demonstrated in AtT20 and GH3 cells, but not in a group of other cell lines: HEK293, PC12, N2a, CATH.a) [44].

On the other hand, CART inhibited the follicle-stimulating hormone (FSH)-stimulated ERK1/2 and Akt signaling in bovine granulosa cells, reducing the kinase activation in a time-dependent manner [45].

#### 3.2.2. CART in Cell Proliferation

The effect of CART on cell proliferation was confirmed by increased cyclin D1 protein expression in CART-treated GLUTag cells (murine enteroendocrine cancer cell line) [46]. As mentioned by Sathanoori et al. [43], the exposure to CART modulates cell proliferation and survival; it induces the phosphorylation of p44/42 MAPK at Thr-202/Tyr-204, FoxO1 at Ser-256, p90^RSK^ (a downstream target of p44/42 MAPK) at Ser-380, and the phosphorylation of CREB and IRS proteins. CART exposure also increases cAMP levels, as shown in β-cells. CART-mediated proliferation in INS-1(832/13) cells was prevented by the addition of pharmacological inhibitors of PKA, Akt and MEK1. Thus, CART, via enhanced cAMP production, seems to regulate downstream effectors, including PKA and MAPK [43]. Both effects promote cellular survival and proliferation since MAPK p44/42 signaling inhibition enhances proteasome inhibitor-mediated apoptosis [47], while cAMP enhances the neuregulin-dependent proliferation of Schwann cells [48].

The data presented above indicate that CART may promote tumorigenesis via multiple pathways. Correspondingly, CART levels are also elevated in various cancer types, both in patients’ plasma and in the tumor cells themselves, vide below.

#### 3.2.3. CART Activity in Breast Cancer

Five publications discussed the association of CART and different types of breast cancer.

It has been shown that *CARTPT* mRNA is expressed in cytological specimens acquired during duct-washing cytology (DWC) derived from solid papillary carcinomas (SPC), a subtype of ductal carcinoma in situ (DCIS), and in intraductal papillomas. Jikuzono et al. [49] chose *CARTPT* and breast cancer-associated transcript 54 (*BRCAT54*) mRNA as markers of quality and quantity of RNA extracted from DWC in the diagnosis of DCIS. DWC samples (12 of 37) were characterized with an RNA integrity of equal or more than 6, representing moderate to high quality. Then, five samples (three malignant, two benign) of DCIS with high RNA yields were elected for quantitative RT-PCR analysis of *CARTPT* and *BRCAT54*. mRNA of *CARTPT* and *BRCAT54* were detectable in all the samples subjected to quantitative RT-PCR, as well as the mRNA of *HPRT-1* (hypoxanthine phosphoribosyl transferase 1, a housekeeping gene), although the *CARTPT*/*HPRT-1* ratio varied from 0.1 to 14.2 in the few samples measured (values from 0.1, 0.1, 0.2, 2.8 to 14.2). Since the measured markers were detected in all samples, RNA from DWC *CARTPT* mRNA was implicated as a potential biomarker of DCIS [49].

Lu et al. [50] compared two histological types of breast cancer: mucinous adenocarcinoma (MC) and invasive ductal carcinoma. Data concerning clinico-pathological features of 186 497 patients were obtained from the Surveillance, Epidemiology, and End Results Program (SEER) 18 database. The mean age of MC patients was higher, with a lower tumor N and G stage value. The proportion of MC cases with positive estrogen receptor (ER) and progesterone receptor (PR) was higher, but it was lower in the case of positive human epidermal growth factor receptor 2 (HER2). Mucinous adenocarcinoma patients received chemotherapy or radiotherapy, and they had a mastectomy less often. Genomic and transcriptomic analysis of 801 cases of IDC or mucinous adenocarcinoma was conducted based on data from The Cancer Genome Atlas (TCGA) database. *CARTPT* expression was found to be upregulated in mucinous adenocarcinoma cases together with *MUC2* and *TFF1* genes [50].

In another article by Brennan et al. [51] the influence of CART on ER-positive, lymph node-negative breast cancer was investigated, together with the possibility of using CART in the diagnosis of this type of tumor. Initially, immunochemistry performed on 10 single-patient tissue microarrays showed that CART protein was expressed in 20% of cases. Expression was restricted to tumor cells and was not evident in normal breast epithelium. Then, after transfection of MCF-7 breast cancer cells with a CMV-CART construct (*CART* cDNA with a pCMV6-XL5 plasmid vector), it was observed that CART stimulates the expression of CART in other cells in an autocrine and paracrine way. CART was also found to be a stimulant of the ligand-independent activation of ER alpha by MAPK-mediated phosphorylation of ER alpha at S188. Finally, in three independent cohorts of ER-positive, lymph node-negative breast cancer, the prognostic factor of CART was assessed. It was shown that high CART expression is associated with decreased overall survival in lymph-node-negative cancer. Moreover, CART is a predictor of the outcome in tamoxifen-treated patients. Increased CART expression was correlated with an impaired response to tamoxifen. CART also protects the breast cancer cell line from tamoxifen-mediated death [51].

In the study by Slattery et al. [52], several energy homeostasis genes were evaluated to find associations of body size measures and breast cancer risk in 3592 cases of breast cancer and 4182 healthy women of either Hispanic/Native American or non-Hispanic white origin. The study also included analysis of five selected single nucleotide polymorphisms (SNPs) of the *CARTPT* gene. The adaptive rank truncated product (ARTP) method revealed that *CARTPT* was associated with the risk of pre-menopausal breast cancer in all women (*P*_ARTP_ = 0.014) and in the group of low Indigenous American (IA) ancestry (*P*_ARTP_ = 0.015). Interestingly, a similar correlation was found for ghrelin prepropeptide in pre-menopausal women of low IA ancestry (P_ARTP_ = 0.007) [52]. 

A similar study was conducted by Rodríguez-Valentín et al. [53]. The aim of their report was to check whether chosen energy homeostasis genes (including the *CARTPT* gene) may modify the association between serum levels of insulin-like growth factor 1 (IGF-1) and insulin-like growth factor binding protein 3 (IGFBP-3) and the risk of breast cancer (BC). The population of the study was limited to pre-menopausal women and included 265 cases of breast cancer and 437 healthy women. DNA was extracted from blood samples, and the serum levels of IGF-1 and IGFB-3 were measured. The participants were divided into tertiles based on IGFBP-3 serum levels. No influence of the *CARTPT* gene on the association between IGF-1 serum level and BC risk was found. There was no association between IGFBP-3 serum level and *CARTPT* rs3846659 SNP. However, *CARTPT* rs3846659 presented to be a significant modifying factor for the association between IGFBP-3 and the risk of BC. In women with dominant homozygotes in the *CARTPT* SNP rs3846659—that is, with the GG genotype—the risk of developing breast cancer was shown to be increased when comparing the highest and the lowest tertile of IGFBP-3 serum levels. No effect was observed in the presence of the minor allele C. These observations show that the *CARTPT* gene polymorphism modifies the association between IGFBP-3 and BC risk [53]. 

#### 3.2.4. CART Activity in Neuroendocrine Tumors

Connections between CART and neuroendocrine tumors were investigated in four articles.

Ramachandran et al. [54] compared the usefulness of CART, chromogranin A (CgA) and chromogranin B (CgB) as biomarkers of various NETs, facilitating the diagnosis and judgment of disease progression. The investigated cancer types included pancreatic, gut, phaeochromocytoma/paraganglioma, nongastroenteropancreatic and unknown primary tumors. The levels of all three peptides were measured in the plasma of 481 confirmed NET patients and compared with control samples from 40 healthy volunteers. The results showed that CART is more useful than both CgA and CgB in the detection of stable and progressive phaeochromocytomas/paragangliomas. This could make CART a surveillance marker for patients with these types of NETs. It was also confirmed that CART is a reliable marker for the identification of progressive pancreatic NETs [54].

Bech et al. [55] investigated a range of neuroendocrine malignancies: gastric, midgut, hindgut, pancreatic, unknown primary, pulmonary, thymic, paraganglionic, ovarian, and renal. CART levels were studied as a tumor marker of neuroendocrine malignancy in the plasma samples from 131 patients. CART levels were also measured in twenty-seven patients after removal of NETs, six patients with newly diagnosed pituitary tumors, thirty-four with various non-neuroendocrine tumors, 153 prostate cancer patients and seventeen patients with renal impairment, in order to investigate whether the measurement of CART levels facilitates identification of nonfunctioning NETs. The control group consisted of 192 patients with other conditions. The plasma CART levels of the vast majority (95%) of patients with progressive pancreatic NETs were increased, with the mean plasma CART levels significantly higher (*p* = 0.035) in those suffering from progressive disease in comparison to patients with all types of stable NETs. When measuring CART and CgA together, their combined sensitivity for discovering neuroendocrine malignancies was higher than the combined sensitivity of CgA and CgB. This shows that CART is a better biomarker than CgB [55].

Landerholm et al. [56] focused on establishing whether CART is expressed in various types of neuroendocrine tumors and, if so, examined CART-expressing cells. Specimens from gastric, ileal, rectal carcinoids, endocrine pancreatic tumors and medullary thyroid carcinomas were used for histopathological examinations and immunohistochemistry. CART-IR (CART immunoreactivity) was found in 80% of all examined NETS; however, its intensity varied between cells. Origin of the neoplasm did not influence CART expression. Either no differences were observed between primary tumors and metastases, or no co-expression of CART and Ki-67 (a cell proliferation marker) was detected [56].

In another study, Landerholm et al. [46] evaluated the role of CART in small bowel carcinoid tumors in a group of 97 patients by histopathological examination and immunohistochemistry. CART expression and higher expression patterns were associated with worse survival. However, CART expression did not correlate with the patients age, disease stage, tumor grade or any manifestation of disease. Next, the effect of CART on tumor cell survival was assessed using GLUTag and HCT-116 cells. CART increased cell viability in both cell lines. In GLUTag cells, an increased cyclin D1 protein expression was observed following CART-treatment [46]. Since cyclin D1 promotes cell cycle progression, CART may stimulate cell division and thus trigger carcinogenesis.

#### 3.2.5. CART Activity in Other Neoplasms

Two articles investigated the role of the *CARTPT* gene in the development of other cancer types.

In the publication by Wang et al. [57] the correlation between variations of the *CARTPT* gene and susceptibility to glioma in the Chinese population was evaluated. The stage I study included 400 patients with a glioma and 400 healthy controls. Four SNPs were selected, and their associations with glioma risk were assessed. Two variants were associated with an increased glioma susceptibility—rs2239670 and rs11575893. Variants rs3846659 and rs6894772 did not display any correlations with the risk of developing glioma. These results were further validated based on 800 glioma cases and 800 healthy controls. Finally, a statistically significant association between rs2239670 (OR = 1.27; 95% CI = 1.10–1.46; *p* = 0.001) and rs11575893 (OR = 1.25; 95% CI = 1.09–1.45; *p* = 0.002) with a higher glioma risk was confirmed in the pooled patient and control groups [57].

Zhou et al. [58] used datasets from TCGA to explore the tumor microenvironment-related genes in cases of stomach adenocarcinoma. The *CARTPT* gene was associated with survival rate—high gene expression correlated with worse survival probability. This was confirmed using data from the Gene Expression Omnibus database [58].

**Table 1:** Patient information from the included human studies.

Study Participants		Scientific Design	References
Case (*n*; M/F ^1^)	Control (*n;* M/F ^1^)		
81; no data	16; no data	Case—CART present, control—no CART	[46]
4 *	1 *	Positive control performed of *BRCAT54*, *CARTPT* and *HPRT-1* gene expression	[49]
801 *	0	Aim—investigation of clinicopathological and genomic features of MC by comparing with IDC	[50]
46 *	272 *	Cohort I; case—high CART, control—low CART	[51]
61 *	311 *	Cohort II; case—high CART, control—low CART	[51]
25 *	25 *	Cohort III; case—high CART, control—low CART	[51]
3592 *	4182 *	Data from the Breast Cancer Health Disparities Study (4-CBCS, MBCS, SFBCS)	[52]
265 *	437 *	Data from the Breast Cancer Health Disparities Study (4-CBCS, MBCS)	[53]
481; 242M/239F	40; no data	Case—NET patients, control—healthy volunteers	[54]
131; no data	192; 100M/92F	Case—NET patients; control—patients with other conditions/complaints	[55]
133; no data	0	Aim—to establish whether CART is expressed in NETs and, if so, the frequency, distribution and phenotype of CART-expressing cells	[56]
400; 244M/156F	400; 248M/152F	Stage I; case—glioma patients, control—healthy subjects	[57]
800; 480M/320F	800; 468M/332F	Stage II; case—glioma patients, control—healthy subjects	[57]
380; 243M/137F	0	Aim—to find the possible prognosis-related genes in STAD using bioinformatics analysis (TCGA database)	[58]
1209M/852F	816M/576F	Total M/F from available data	
4794	5228	Total BC study participants (F)	
345	56	Total patients with gender unspecified	
7200	6676	Total number of patients	

4-CBCS—4-Corner’s Breast Cancer Study, IDC—invasive ductal carcinoma, MBCS—Mexico Breast Cancer Study, MC—mucinous adenocarcinoma, NET—neuroendocrine tumor, SFBCS—San Francisco Bay Area Breast Cancer Study, STAD—stomach adenocarcinoma, TCGA—The Cancer Genome Atlas, ^1^ where applicable, * breast cancer study, only females.

#### 3.2.6. CART Activity in Cancer Cell Lines

Four publications described the influence of CART on cancer cell lines—rat adrenal pheochromocytoma—PC12 [59,60], rat insulinoma—INS-1(832/13) [43] and murine pituitary tumor—AtT-20 [44].

**Table 2:** Publications included by full text describing the role of CART in different types of cancer.

Type of Neoplasm	Study Goal	Methods	Main Outcomes	References
Breast cancer at early stage.	RNA recovery quantification from duct-washing cytology specimens of DCIS, using *CARTPT* and *BRCAT54* mRNAs.	45 specimens for DWC. *CARTPT*, *BRCAT54*, and *HPRT-1* mRNAs analyses via qRT-PCR. qRT-PCR analysis of five samples with high RNA yields (three malignant, two benign) for *CARTPT* and *BRCAT54*. Positive control—SPC breast tumor tissue RNA.	(1) RNA of acceptable quality and quantity can be obtained from DWC specimens and used in RNA-based pre-operative diagnosis of breast carcinoma. (2) CART may serve as a biomarker of DCIS as *CARTPT* mRNAs were detected in all samples (as for *HPRT-1* mRNA—a representative housekeeping gene.	[49]
Breast cancer Mucinous adenocarcinoma (MC); Invasive ductal carcinoma (IDC).	Comparison of clinicopathological and genomic characteristics of MC with IDC.	186,497 patients (4578 MC and 181,919 IDC) data from SEER registry (18 databases 1973–2015). Patient characteristics: age at diagnosis, race, grade, ER, PR and HER2 status, T and N stage, radiation, chemotherapy and surgery type. Gene set enrichment analysis. 801 IDC/MC cases from TCGA cohort—genomic and transcriptomic analysis, including *CARTPT*.	(1) *CARTPT*, *MUC2* and *TFF1* genes upregulated in MC. (2) Neurotransmitter release-related pathways (including GABA) upregulation and amplification of 6p25.2, 6q12 and 11q12.3 in MC. (3) MC cases—older, lower G and N tumor stage, higher % of positive ER and PR, lower % of positive HER2 and less cases treated with radiation, chemotherapy and mastectomy compared to IDC.	[50]
Breast cancer ER-positive, lymph node-negative.	Search for prognostic and predictive biomarkers of ER-positive, lymph node-negative breast cancer.	Immunohistochemistry—10 single patient TMA with normal breast epithelium, invasive cancer or lymph node metastasis. Transfection of MCF-7 cells with CMV-CART construct—Western blot. Treatment of MCF-7 cells with recombinant human CART_42–89_ peptide—Western blot, Luciferase reporter assay. Manual cytoplasmic intensity score and automated image analysis—CART protein expression in two independent breast cancer cohorts. MCF-7 and T47D cells (response to tamoxifen measurement)—fluorescence-activated cell sorting analysis. 3rd cohort—patients under tamoxifen treatment.	(1) CART is expressed in primary and metastatic breast cancer. (2) CART stimulates cells by an autocrine and paracrine loop. (3) CART expression leads to the activation of MAPK pathway. (4) CART increases the ligand-independent activation of ERα through MAPK-mediated phosphorylation of ERα at S118. (5) CART is an independent poor prognostic factor in ER-positive lymph node negative tumors. (6) Ectopic CART expression in ER positive breast cancer cell lines promotes survival in tamoxifen treated cells. (7) High CART expression was associated with poor response to tamoxifen.	[51]
Breast cancer	Assessment of link between 10 energy homeostasis genes (including *CARTPT*) and breast cancer risk in connection with ethnical ancestry, body size measurements and menopausal status.	2111 cases and 2579 controls in a Hispanic/Native American population and 1481 cases and 1585 controls in a non-Hispanic white population. Data from the Breast Cancer Health Disparities Study, the 4-Corners Breast Cancer Study, the Mexico Breast Cancer Study and the San Francisco Bay Area Breast Cancer Study. DNA collected from blood or mouthwash samples—whole genome amplification and SNP analyses of 10 genes including *CARTPT* (5 SNPs) expression	(1) Association between the *CARTPT* gene and the risk of breast cancer in a group of pre-menopausal women with low Indigenous American ancestry and in the group of all pre-menopausal women.	[52]
Breast cancer	Analyses of modifying effect of 10 energy homeostasis genes (including *CARTPT*) on the association between IGF-1 and IGFBP-3 serum levels and the risk of BC.	265 Premenopausal breast cancer cases and 437 controls. *CARTPT* expression IGF-1 and IGFBP-3 serum levels.	(1) No effect of *CARTPT* on the association of IGF-1 serum level and BC risk. (2) No association between IGF-1 serum level and *CARTPT*. (3) GG genotype of *CARTPT* rs3846659 SNP increased the risk of BC in the group of patients with IGFBP-3 serum level in the highest tertile. (4) The presence of the C allele eliminated this effect.	[53]
Neuroendocrine tumors (NETs): Pancreatic; Gut; PCC/PGL; NonGEP; Unknown primary.	To evaluate the comparative and combined utility of CART, CgA and CgB for diagnosis and monitoring of different NET subtypes.	A total of 481 samples from confirmed NET cases and 40 controls. Measurement of plasma CART, CgA and CgB levels with radioimmunoassay. Retrospective collection of clinical data. Exclusion—renal insufficiency (eGFR ≤ 60 mL/min/1.73 m^2^)	(1) CART—marker for the identification of progressive pancreatic NETs; superior to CgA and CgB in detecting stable and progressive PCC or PGLs. (2) CART plasma levels decreased after resection of tumors, remained low in all patients in remission, but increased when progression of the disease occurred. (3) The measurement of CART did not improve diagnosis in gut, nonGEP NETs, and NETs with an unknown primary origin in comparison with combined CgA and CgB measurement.	[54]
NETs: Midgut; Pancreatic; Unknown primary; Hindgut; Pulmonary; Thymic; Paraganglionic; Gastric; Ovarian; Renal.	To investigate the potential role of plasma CART levels as a biomarker of neuroendocrine malignancies.	Twelve volunteers—analysis of fasting effect on plasma CART levels. A total of 131 patients with neuroendocrine tumors: fifty-one midgut, thirty-eight pancreatic, five hindgut, five pulmonary, two thymic, two paraganglionic, two gastric, one ovarian, one renal, twenty-four of unknown origin. Sixty-eight patients with stable, sixty-three with progressive illness. Measurement of plasma CgA, CgB and CART immunoreactivity levels (gel chromatography and reverse-phase fast protein liquid chromatography).	(1) A measure of 150 pmol/L—the upper limit of normal CART levels. (2) NET patients—mean plasma CART 440 pmol/L; elevated in 56% of patients. (3) CART levels with chromogranin A measurement—improved sensitivity for neuroendocrine malignancy from 85% to 91%; the sensitivity of CgA and CgB—89%. (4) Increased CART levels—pancreatic tumor (71% of all patients, 95% of patients with progressive disease), midgut tumor (45%), tumor of unknown origin (63%). (5) Mean CART levels in plasma were higher in progressive diseases than in stable tumors of all types.	[55]
NETs: Gastric carcinoids; Ileal carcinoids; Rectal carcinoids; Endocrine; Pancreatic tumors; Medullary thyroid carcinomas.	Expression of CART in different types of NETs and analysis of the frequency, distribution and phenotype of cells expressing CART.	Paraffin-embedded specimens from: gastric carcinoids (28), ileal carcinoids (58), rectal carcinoids (15), EPT (9), MTC (23). CART expression (immunohistochemistry and in situ hybridization) Control—scattered background labelling when using a 100-fold excess of unlabeled probe.	(1) In the majority of examined NETs, tumor cells with the expression of CART were found. (2) CART was located in a major population of tumor cells in 14% of NETs. (3) CART expression pattern was heterogeneous between different types of tumors and within individual tumors as well. (4) CART may be used in diagnosing NET specimens in histopathological examinations.	[56]
Small bowel carcinoid	To assess link between CART expression and survival rates in patients with small bowel carcinoid or between CART expression and small bowel carcinoid characteristics/clinical symptoms.	Ninety-seven jejunal, ileal or ileocecal valve carcinoid specimens. CART expression using immunohistochemistry. Clinical data collection from medical records. GLUTag and HCT-116 cell lines—assessment of the influence of CART on cell viability.	(1) CART expression associated with worse survival rate (specific to the disease). (2) Adjusting for age, disease stage and grade gave the same results. (3) CART expression was not associated with any of presenting symptoms, disease stage, tumor grade or age. (4) In vitro—CART promoted viability of GLUTag and HCT-116 tumor cell lines by increasing proliferation.	[46]
Stomach adenocarcinoma (STAD)	Study of a prognostic value of tumor microenvironment-related genes in STAD.	A total of 380 patients with STAD. RNA-seq data from TCGA database and *CARTPT* gene expression measurements. Clinical data from cBioportal website. GSE84433 expression dataset and clinical data from GEO database of 357 samples.	High *CARTPT* gene expression was associated with poor overall survival in patients with STAD.	[58]
Glioma	To investigate the association between genetic polymorphisms in the *CARTPT* gene and glioma risk in the Chinese population.	1st stage—400 cases, 400 controls; 2nd stage—800 cases, 800 controls. *CARTPT* gene analysis—CART tag SNPs (rs2239670, rs3846659, rs11575893, rs6894772) DNA from blood samples; SNP genotyping	(1) The rs2239670 (genotype AA) and rs11575893 (genotype CT and TT) variants of the *CART* gene were associated with increased glioma susceptibility.	[57]
PC12 cell line	To characterize CART binding to PC12 cells.	Iodination of CART_61–102_ at Tyr-62. Mice—study of the influence of iodination on the biological function of CART. Assessment of ^125^I-CART_61–102_ binding to differentiated PC12 cells.	(1) Di-iodinated CART_61–102_ is biologically active. (2) CART binds specifically to PC12 cells, both to intact cells and cell membranes. (3) Specific binding sites were found on non-differentiated and differentiated cells.	[59]
PC12 cell line	To study the effect of differentiation of PC12 cells on CART binding and CART-activated signaling;Characterization of the pharmacological profile of CART binding sites.	CART_61–102_ iodination at Tyr62. ^125^I-CART_61–102_ binding on intact PC12 cells and cell membranes. Western blotting (anti-p-ERK and anti-ERK primary antibodies). [^35^S]-GTPγS binding assay.	(1) Differentiation led to increase in specific binding of CART. (2) CART binding sites in PC12 seem to be G-protein coupled receptors linked with Gi/o. (3) CART_61–102_ stimulates the phosphorylation of ERK. (4) PACAP_6–38_ is an effective CART receptor antagonist.	[60]
INS-1(832/13) β-cell line and isolated rat islets	To evaluate the role of CART in β-cell viability and functioning and to investigate its signaling pathways.	INS-1(832/13) β-cell culture. *CART* mRNA expression determined by RT-quantitative PCR. CART protein measurement by Western blot.	(1) Reduced expression of endogenous CART in the INS-1 (832/13) cells at 25 mm glucose (2) CART regulates secretion of insulin, promotes cells proliferation and viability. (3) CART reduces glucotoxicity in β-cells INS-1 832/13. (4) CART signaling is induced by activation of cAMP, CREB, IRS, PKB (Akt) and FoxO1, p44/42 MAPK and p90^RSK^.	[43]
Cell lines: AtT20; GH3; HEK293; PC12; N2a; CATH.a (rat pituitary cell lines).	To evaluate the effect of CART_55–102_ on the MAPK cascade and the phosphorylation state of ERK1 and 2 in a pituitary-derived cell line.	Cell line cultures. Exposure to CART_55–102._ Measurement of levels of phosphorylated ERKs.	(1) The largest effects induced by CART on ERK phosphorylation state in AtT20 cells (2) CART_55–102_ activates ERK1 and 2 (time- and dose-dependently). (3) CART effect is blocked by U0126—involvement of MEK1 and 2. (4) Pertussis toxin inhibits P-ERK formation in response to CART—possibility of a Gi/o coupled receptor (GPCR).	[44]

AtT20—mouse pituitary gland tumor cells, BC—breast cancer, cAMP—cyclic adenosine monophosphate, CART—cocaine- and amphetamine-regulated transcript, CART-IR—CART immunoreactivity, CARTPT—cocaine- and amphetamine-regulated transcript prepropeptide, CATHa—mouse locus coeruleus cell line, CgA—chromogranin A, CgB—chromogranin B (CgB), CREB—cAMP response element-binding protein, DCIS—ductal carcinoma in situ, DWC—duct washing cytology, EPT—endocrine pancreatic tumor, ER—estrogen receptor, ERK—extracellular signal-related kinase, FoxO1—forkhead box protein O1, GABA—γ-aminobutyric acidGH3—rat pituitary cell line, GLUTag—murine glucagon-producing enteroendocrine tumor, GPCR—Gi.Go coupled receptor, HCT-116—human colon cancer, HEK293—human epithelial embryonic kidney cells, HER2—human epidermal growth factor receptor-2, IDC—invasive ductal carcinoma, IGF-1—insulin-like growth factor-1, IGFBP-1—insulin-like growth factor binding protein, INS-1(832/13)—rat insulinoma cell line, IRS—insulin receptor substrate protein, MAPK—mitogen-activated protein kinase, MC—mucinous adenocarcinoma, MCF-7—human breast cancer cell line, MEK1—mitogen-activated protein kinase kinase, MTC—medullary thyroid carcinoma, N2a—mouse neuroblastoma cell line, NET—neuroendocrine tumor, NGF—nerve growth factor, nonGEP—nongastroenteropancreatic, p90^RSK^—90 kDa ribosomal S6 kinasePACAP—pituitary adenylate cyclase-activating polypeptide, PC12—rat pheochromocytoma cell line, PCC/PGL—phaeochromocytomas/paragangliomas, PKB—protein kinase B, PR—progesterone receptor, qRT-PCR—real-time quantitative reverse transcription PCRRIN—RNA integrity number, SNP—single-nucleotide polymorphism, SPC—solid papillary carcinomas, STAD—stomach adenocarcinoma, T47D—human breast cancer cell line, TCGA—The Cancer Genome Atlas, TMA—tissue microarrays, TUNEL—terminal deoxynucleotidyl transferase (TdT)-mediated biotinylated UTP nick end labeling, U0126—1,4-diamino-2,3-dicyano-1,4-bis[2-aminophenylthio]butadiene.

**Table 3:** Publications included by full text describing signaling pathways involved in cell cycle regulation, proliferation and apoptosis.

Pathway	Methods	Results	References
RAF → MEK → ERK PI3K → PDK1 → AKT ERK + AKT → promotion G0/G1/S-phase entry	Cell cultures: NIH 3T3 (mouse embryo fibroblasts),NIH 3T3:iRAS (NIH 3T3 cells with HRAS^G12V^ expression), MEFs (mouse embryo fibroblasts). Immune complex kinase assays. Western blotting. DNA synthesis assays. QT-PCR, RNA protection assays. Confocal immunofluorescence microscopy.	(1) Coactivation of RAF and AKT increases cell proliferation; kinases cooperatively induce cyclin D1 and repress p27^Kip1^ expression. (2) High level of RAF activation leads to G1 cell cycle arrest; effect is blocked by activation of Akt. (3) MEK and PI3K pathways are necessary for repression of p27^Kip1^. (4) Activation of Akt stimulates RAF-induced relocation of p21^Cip1^ from CDK2 complexes.	[42]
acute CART stimulation → receptor → G_o/i_ → PKC→ MEK → activation of ERK1/2 → expression of DUSP5 → inhibition of ERK CART → impaired proteasomal degradation + increased synthesis → increased activity of DUSP5 and PP2A → inhibition of ERK and Akt pathways	Bovine granulosa cells culture. Western blotting. In vitro phosphatase assay. RNA interference in vitro.	(1) CART stimulation inhibits FSH-induced ERK1/2 and Akt signaling. (2) Kinase inhibition is mediated by increased activity of DUPS5 and PP2A due to promotion of their synthesis and inhibition of proteasomic degradation. (3) A negative feedback loop—acute CART stimulation leads to activation of ERK1/2 via Gi/o, PKC and MEK, but later to DUSP5 expression which stops in turn ERK1/2.	[45]
Proteasome inhibitors → induction of MPK → dephosphorylation of p44/42 MAPK → apoptosis	Cell cultures: A1N4-myc (human mammary epithelial cells), MDA-MB-231 (breast adenocarcinoma cells), BT-474 (breast ductal carcinoma cells). Western blotting. Apoptosis assays (ELISA).	(1) Inhibition of proteasome leads to decrease in activated p44/42 MAPK and induction of MKP. (2) MAPK dephosphorylation by proteasome inhibitors was mediated by phosphatases—mostly by MKP-1. (3) Proteasome inhibitors triggered inhibition of p44/42 MAPK kinase which eventually led to apoptosis.	[47]
Neuregulin → cAMP-mediated pathways → G1-S progression cAMP → RAF/MEK → ERK cAMP → PDK → PI3K/Akt	Primary human and rat Schwann cell cultures. Proliferation assays. Western blotting. Immunofluorescence microscopy.	(1) Activators of adenylyl cyclase and increased cAMP levels reduce S-phase initiation time. (2) cAMP sustains neuregulin-induced MEK-ERK activity and enhances neuregulin-induced PI3K/Akt kinase activity. (3) Prolonged activity of MEK/ERK and PI3K/Akt leads to S-phase entry.	[48]

Akt—protein kinase B, cAMP—cyclic adenosine monophosphate, CART—cocaine- and amphetamine-regulated transcript, CDK2—cyclin dependent kinase 2, DUSP5—dual specific phosphatase 5, ELISA—enzyme-linked immunosorbent assay, ERK 1/2—extracellular regulated kinase 1/2, FSH—follicle- stimulating hormone, MAPK—mitogen-activated protein kinase, MEK—mitogen-activated protein kinase kinase, MPK—dual specificity MAPK phosphatase, PDK—phosphoinositide-dependent protein kinase, PI3K—phosphatidylinositol 3′-kinase, PKC—protein kinase C, PP2A—protein phosphatase 2A, RT-qPCR—quantitative reverse transcription-polymerase chain reaction, RAF—rapidly accelerated fibrosarcoma.

Maletínská et al. [59] investigated the binding of iodinated CART peptide (^125^I-CART_61–102_) to PC12 cells. Those were both non-differentiated as well as treated with nerve growth factor (NGF) and developed into neuronal phenotype. It was shown that ^125^I-CART_61–102_ binds specifically to both investigated cell types; however, the binding capacity of differentiated cells was higher than of those non-differentiated. CART was bound to intact cells, and isolated membranes as well. The results suggested the presence of CART receptors (not known at that time) on PC12 cells and cells derived from them [59]. 

Indeed, Lin et al. [60] later investigated the pharmacological profile of CART binding sites on the PC12 cell line and the association between increased CART binding and CART-activated signaling in PC12 cells after their neuronal differentiation. Before differentiation, CART’s influence was small but significant, causing an increase in ERK phosphorylation. The addition of CART_61–102_ to NGF-treated cells produced a much more considerable increase in phospho-ERK (p-ERK), demonstrating that CART-activated signaling intensifies with differentiation. Similarly, an increase in CART binding capacity occurs with differentiation of PC12 cells. A GPCR coupled to Gi/o can mediate CART signaling in these cells [60].

In the publication by Sathanoori et al. [43], the effect of exogenous CART_55–102_ on β-cell viability was judged. The signaling mechanisms of this peptide were also investigated. Studies on the INS-1(832/13) clonal β-cell culture and isolated rat islets showed that CART decreased glucotoxicity-induced apoptosis in the INS-1(832/13) cells and β-cell apoptosis in the rat islets (by 63% and 66%, respectively) [43].

Lakatos et al. [44] revealed that CART_55–102_ activates ERK1 and 2 in a time- and dose-dependent manner in AtT20 cells, and that this effect is absent in the presence of U0126, a highly selective MEK1 and 2 inhibitor, demonstrating an involvement of these kinases in the signaling pathway. Since pertussis toxin, a Gi and Go signaling inhibitor, hindered p-ERK formation, the involved receptor is likely to be a Gi/o coupled GPCR. Genistein, an inhibitor of receptor tyrosine kinase signaling, did not exert such effects [44].

## 4. Conclusions

The results of the studies included in this systematic review suggest that CART could be a potential valuable diagnostic biomarker in various types of oncological diseases, which may lead to the improvement of early cancer detection. There is an increasing amount of evidence showing the potential role of CART in the modulation of neoplastic processes. Therefore, further experimental and clinical research is needed to clarify the role of CART in cancer pathogenesis. Identifying precise mechanisms of CART action may result in the development of new therapeutic approaches and the design of novel antineoplastic agents.

## Data Availability

Not applicable.
